# Biochemical and molecular changes associated with heteroxylan biosynthesis in *Neolamarckia cadamba* (Rubiaceae) during xylogenesis

**DOI:** 10.3389/fpls.2014.00602

**Published:** 2014-11-07

**Authors:** Xianhai Zhao, Kunxi Ouyang, Siming Gan, Wei Zeng, Lili Song, Shuai Zhao, Juncheng Li, Monika S. Doblin, Antony Bacic, Xiao-Yang Chen, Alan Marchant, Xiaomei Deng, Ai-Min Wu

**Affiliations:** ^1^Guangdong Key Laboratory for Innovative Development and Utilization of Forest Plant Germplasm, South China Agricultural University, GuangzhouChina; ^2^State Key Laboratory for Conservation and Utilization of Subtropical Agro-bioresources, GuangzhouChina; ^3^State Key Laboratory of Tree Genetics and Breeding, Chinese Academy of Forestry, BeijingChina; ^4^Research Institute of Tropical Forestry, Chinese Academy of Forestry, GuangzhouChina; ^5^ARC Centre of Excellence in Plant Cell Walls, School of Botany, The University of Melbourne, Parkville, VICAustralia; ^6^Nurturing Station for the State Key Laboratory of Subtropical Silviculture, Zhejiang A & F University, HangzhouChina; ^7^Bio21 Molecular Science and Biotechnology Institute, The University of Melbourne, Parkville, VICAustralia; ^8^Centre for Biological Sciences, University of Southampton, SouthamptonUK; ^9^College of Forest, South China Agricultural University, GuangzhouChina

**Keywords:** heteroxylan, *Neolamarckia cadamba* (Rubiaceae), RNA-seq, XylT activity, xylogenesis

## Abstract

Wood, derived from plant secondary growth, is a commercially important material. Both cellulose and lignin assembly have been well studied during wood formation (xylogenesis), but heteroxylan biosynthesis is less well defined. Elucidation of the heteroxylan biosynthetic pathway is crucial to understand the mechanism of wood formation. Here, we use *Neolamarckia cadamba*, a fast-growing tropical tree, as a sample to analyze heteroxylan formation at the biochemical and molecular levels during wood formation. Analysis of the non-cellulosic polysaccharides isolated from *N. cadamba* stems shows that heteroxylans dominate non-cellulosic polysaccharides and increase with xylogenesis. Microsomes isolated from stems of 1-year-old *N. cadamba* exhibited UDP-Xyl synthase and xylosyltransferase activities with the highest activity present in the middle and basal stem regions. To further understand the genetic basis of heteroxylan synthesis, RNA sequencing (RNA-seq) was used to generate transcriptomes of *N. cadamba* during xylogenesis. The RNA-seq results showed that genes related to heteroxylan synthesis had higher expression levels in the middle and basal part of the stem compared to the apical part. Our results describe the heteroxylan distribution and heteroxylan synthesis trait in *N. cadamba* and give a new example for understanding the mechanism of heteroxylan synthesis in tropical tree species in future.

## INTRODUCTION

Wood formation (xylogenesis) is a critical developmental process for all woody land plants and is important for mechanical support as well as water and mineral transport. Wood formation is also a good model system to study plant cell wall biosynthesis at the DNA, RNA, and protein levels (Bailey, 1952; Plomion et al., 2001), but our understanding of xylogenesis is still elementary. Plant cell walls are predominantly composed of cellulose, non-cellulosic polysaccharides, and lignin which represent the most abundant renewable resource on Earth (Pauly and Keegstra, 2008). Cellulose, a β-1,4-glucan, accounts for the highest proportion of the cell wall (Pauly and Keegstra, 2010). Lignin is an intricate biopolymer comprising 4-hydroxyphenyl propanoids. The presence of lignin in the cell wall can prevent cellulose degradation (Simmons et al., 2010). Non-cellulosic polysaccharides account for more than a quarter of the dry mass of cell walls in dicot plants and are widely used in the food and pharmaceutical industries. Despite its importance, the heteroxylan biosynthetic mechanism is not well understood.

Heteroxylan is the major non-cellulosic polysaccharides in the secondary walls of dicot plants with a linear β-1,4-Xyl backbone. According to the nature of the side chains, heteroxylan is designated as glucuronoxylan (GX), 4-*O*-methylglucuronoxylan (MGX), arabinoxylan (AX), and glucuronoarabinoxylan (GAX). The Xyl residues of the heteroxylan backbone can also be substituted with *O*-acetyl groups at various *O*-positions (Gille and Pauly, 2012). In addition to the side chain substitutions, a reducing end tetrasaccharide β-D-Xyl-(1, 3)-α-L-Rha-(1, 2)-α-D-GalA-(1, 4)-D-Xyl has been found as part of the GX of dicots and gymnosperm species (Peña et al., 2007) but not in grass heteroxylans (Scheller and Ulvskov, 2010; Ratnayake et al., 2014). As heteroxylan comprises a significant proportion of woody tissue, elucidating the mechanism of heteroxylan biosynthesis is important for efficient biomass generation and subsequent utilization.

The biosynthesis of heteroxylan in dicots involves numerous glycosyltransferases (GTs) genes including members of the GT 43, GT 47, and GT 8 families (Doering et al., 2012). It has been proposed that different GT enzymes could form functional protein complexes in order to synthesize heteroxylan (Faik, 2010). IRX9, IRX9L, IRX14, IRX14L (GT 43), IRX10, and IRX10L (GT 47; Lee et al., 2007a; Wu et al., 2009, 2010) comprise three distinct pairs (IRX9 and IRX9L, IRX10 and IRX10L, IRX14 and IRX14L), each differentially contributing to heteroxylan backbone biosynthesis (Wu et al., 2009, 2010; Lee et al., 2010). FRA8, F8H (GT 47), IRX8, and PARVUS (GT 8) may be involved in the synthesis of the heteroxylan reducing end tetrasaccharide (Zhong et al., 2005; Brown et al., 2007; Lee et al., 2007b, 2009; Peña et al., 2007). Glucuronosyl transferases (GlcATs) from the GT 8 family, GUX1, GUX2, GUX3, GUX4, and GUX5 are proposed to function in the addition of either glucuronic acid (GlcA) or 4-*O*-methyglucuronic acid (MeGlcA) to the xylan backbone (Mortimer et al., 2010; Rennie et al., 2012; Bromley et al., 2013). The GX methyltransferase (GMX) contains a DUF579 domain, which has now been proposed to represent a polysaccharide specific *O*-methyltransferase (Lee et al., 2012a; Urbanowicz et al., 2012). Additionally, the plant-specific DOMAIN OF UNKNOWN FUNCTION (DUF) 231 family member ESKIMO1/TBL29 has been reported as a putative heteroxylan *O*-acetyltransferase to be responsible for *O*-acetylation on both *O*-2 and *O*-3 positions of the xylan backbone (Xiong et al., 2013; Yuan et al., 2013).

Heteroxylan is synthesized in the Golgi, and then transported to the plasma membrane via secretory post-Golgi vesicles where it is integrated into the wall. Microsomes isolated from poplar stems contain xylosyltransferase (XylT) activity that adds up to seven Xyl residues to exogenous xylo-oligosaccharide acceptors (Lee et al., 2012c). In other studies, XylT activity in microsomes isolated from wheat seedling and barley endosperm can add up to five Xyl residues to exogenous xylo-oligosaccharide acceptors (Kuroyama and Tsumuraya, 2001; Urahara et al., 2004). Heteroxylan backbone elongation is synthesized by XylT using UDP-xylose as substrate. The synthesis of UDP-Xyl involves UDP-Xyl synthase (UXS; Harper and Bar-Peled, 2002; Pattathil et al., 2005).

In expanding shoots of trees, the apical portion is soft, while the basal portion is hard. This is the result of cell wall thickening and lignification of secondary cell walls. Studies into the transition from primary to secondary growth will be beneficial to understand the process of xylogenesis and heteroxylan formation. Currently, lots of research concerning secondary cell wall formation is based on either cDNA microarray analysis (Hertzberg et al., 2001; Yang et al., 2004) or analysis of proteins isolated from woody tissues (Costa et al., 1999; Vander Mijnsbrugge et al., 2000). In comparison, RNA sequencing (RNA-seq), a recently developed approach based on next-generation sequencing (NGS), enables genetic studies of species without reference to genome sequence information (Brautigam et al., 2011). It has been widely applied in plant biology, such as molecular marker development, transcriptional profiling and gene discovery, both in model species such as *Arabidopsis thaliana* (Loraine et al., 2013; Vidal et al., 2013) and *Nicotiana benthamiana* (Nakasugi et al., 2013) as well as non-model species including *Prunus persica* (Wang et al., 2013) and *Camellia sinensis* (Shi et al., 2011) and tree species (Kullan et al., 2012; Qiu et al., 2013). This technology should also be an efficient approach to understand heteroxylan synthesis in tree species with un-sequenced genomes.

Forest trees represent the dominating biomass production on land and natural forest cannot meet current global wood demand. Artificial forest of fast-growing trees has the capacity to provide the vast needs of wood on a long term. *Neolamarckia cadamba*, a member of *Neolamarckia* tribe in the Rubiaceae family, is distributed widely in south Asia and the south of China (Ouyang et al., 2013). It is reported that, under normal conditions, *N. cadamba* could attain an average height of about 17 m and diameter of 25 cm at breast height within 9 years (Zayed et al., 2014). It is also one of the best raw materials for the plywood industry, pulp, and paper production. Moreover, *N. cadamba* also served as medicinal plant for traditional curing, such as anti-diuretic, treatment of fever and anemia and so on (Ahmed et al., 2011). The bioactivity properties also have been studied, such as antimicrobial, antioxidant, antidiarrheal, and wound healing (Umachigi et al., 2007; Alam et al., 2008). *N. cadamba* has been chosen as one of the artificial tree species in forest rehabilitation projects in south Asia countries due to its short rotation period (Zayed et al., 2014). Although some molecular studies have been initiated in *N. cadamba* for selective breeding (Ho et al., 2014; Tiong et al., 2014) to overcome the long history problem of traditional breeding, along with the demand on biomass accelerating, the effective strategy of molecular breeding becomes urgent.

Although there have been extensive efforts to unravel the genetic regulation of wood formation in trees (Allona et al., 1998; Hertzberg et al., 2001), the biochemistry and molecular events involved in the transition from primary to secondary growth are largely unknown. Intense secondary growth was occurred and accumulated during tree growing and *N. cadamba*, a fast-growing tree in tropical area, can present a good example to enrich the mechanism of secondary cell wall formation by comparing with *Arabidopsis* and model tree populous which grows slower in temperate area. In this report, we investigate the xylem of the 1-year old *N. cadamba* stem using compositional analysis, enzyme activity assays, and gene expression. To gain an understanding of molecular mechanism of heteroxylan synthesis in different parts of the stem, we initiated a RNA-seq project for *N. cadamba* (NCBI Bioproject Accession: PRJNA232616^1^). Our results will provide useful information for wood formation studies and accelerate the molecular breeding on this fast-growing tree.

## MATERIALS AND METHODS

### PLANT MATERIALS AND GROWTH CONDITIONS

*Neolamarckia cadamba* was grown in a greenhouse at 28/24°C, 14/10 h (day/night) and 330 μmol m^-2^ s^-1^ light. Stems used for microscopy, microsome isolation, and compositional analysis were collected from 1-year-old plants with 1.2 m height approximately. The stem was divided into apical, middle, and basal segments each of 20 cm in length.

### SECTIONING OF STEMS

Tissues of the three stem regions were fixed in FAA [5% (v/v) formalin, 5% (v/v) glacial acetic acid, 65% (v/v) ethyl alcohol] for a month. Sections (40 μm) were cut using a Leica VT1000S vibratome supported by 3% (w/v) agarose. The sections were stained in 0.02% (w/v) toluidine blue O (Sigma-Aldrich) for 1–2 min according to (Wu et al., 2010), and then observed with a light microscope (Olympus BX43F).

### HETEROXYLAN IMMUNOLOCALIZATION

Sections were cut using a Leica VT1000S vibratome and immunolocalized following the procedure described below. Sections (40 μm) were incubated with LM10 monoclonal antibodies (1/20 dilution; Plantprobes^2^) for 1 h, and were then washed five times with 0.1 M PBS buffer (0.1 M PBS and 0.5 M NaCl, pH 7.2), followed by incubation for 1 h with fluorescein isothiocyanate-conjugated antibodies (1/50 dilution; Jackson ImmunoResearch Laboratories, Inc.^3^). Following five further washes, the immunofluorescence was observed using a Zeiss LSM710 confocal microscope^4^. For each of the three stem regions, more than seven sections were immunolocalized with LM10 antibody.

### MEASUREMENT OF CONTENT OF CELLULOSE, NON-CELLULOSIC POLYSACCHARIDES, AND LIGNIN

The three stem regions were dried, ground and 40–60 mesh fractions (450–300 μm) were selected to allow chemical composition analysis. Particles graded using a 60 mesh sieve were directly used for the Fourier transform infrared spectroscopy (FT-IR) assay. Dewaxing powders used for the following measurement were extracted with toluene–ethanol (2:1, v/v) in a Soxhlet for 6 h at reflux (∼95°C). De-waxing powders were subjected to sulfuric acid hydrolysis as specified in standard Tappi T222 om-02 for acid-insoluble lignin. The acid soluble lignin can be measured by absorption of ultraviolet radiation [ε205 = 110L (g cm)^-1^]. The de-waxing powders were de-lignified in sodium chlorite (pH 4.0, adjusted using acetic acid, 75°C) for 4 h, leaving holocelluloses (non-cellulosic polysaccharides and cellulose). The content of cellulose was determined by Kurschner–Hoffner’s method. De-waxing powders (1 *g*, dry weight) were hydrolyzed with nitric acid-ethanol several times in boiling water till the fiber whitened. The alcoholic nitric acid solution was discarded and a fresh volume was added after each cycle. The nitric acid-ethanol solution was obtained by mixing one volume of 65% (w/w) nitric acid solution with four volumes of 96% absolute ethyl alcohol. After four cycles, the cellulose was washed, dried and weighed. The difference between the values of holocellulose and cellulose was defined as the non-cellulosic polysaccharide content of the wood powder.

### POLYSACCHARIDE COMPOSITIONAL ANALYSIS OF WHOLE CELL WALLS

Alcohol-insoluble residue (AIR) was prepared and methylation analysis for both neutral and acidic monosaccharide linkage composition was performed following the procedure described by Pettolino et al. (2012). Monosaccharide linkage analysis was performed on a Hewlett-Packard 6890 Gas Chromatograph with a Hewlett-Packard 5973 Mass Spectrometer (Agilent) equipped with a BPX70 column (25 m × 0.22 mm inner diameter, film = 0.25 μm, SGE).

### ISOLATION OF NON-CELLULOSIC POLYSACCHARIDES

The holocellulose was extracted with 2 N KOH with a solid to liquid ratio of 1:20 (g mL^-1^) for 6 h at 50°C. Filtrate was acidified with glacial acetic acid until the pH reached 5.5. The filtrate was then mixed with two volumes of ethanol and the precipitates formed were recovered by filtration and freeze-drying.

### FOURIER TRANSFORM INFRARED

The KBr disk standard technique was utilized in the preparation of wood flour samples for infrared measurements. Briefly, the finer fraction (2 mm) of the mixture of 1 mg sample and 100 mg KBr powders are finely powdered in an agate mortar. Then the mixture was squeezed with a tablet press machine with 10 ton pressure sustained for 1–2 min. FT-IR spectroscopy in transmittance mode was performed on a BRUKER VERTEX 70 spectrometer^5^. The mid-IR spectra ranging from 4000 to 400 cm^-1^ were selected with total of 32 scans at a resolution of 4 cm^-1^.

### PREPARATION OF MICROSOMAL FRACTION

Microsomes were isolated from the apical, middle and basal parts, following the procedure of Kuroyama and Tsumuraya (2001) with minor modification. All subsequent operations were carried out at 0–4°C. About 40 *g* of stem was crushed with a one liter laboratory blender (CAC33^6^) in 150 mL homogenization buffer containing 100 mM Hepes-KOH buffer (pH 6.8), 1 mM DTT, 1 mM EDTA, 0.1 mM MnCl_2,_ and 0.4 M sucrose. The homogenate was filtered through filter gauze and the filtrate was successively centrifuged at 1,000 *g* for 10 min and 10,000 *g* for 10 min. The supernatant was ultracentrifuged at 100,000 *g* for 1 h. The microsomal membrane pellets were rinsed with the homogenization buffer five times and resuspended in buffer (0.5–1 mL) containing protease inhibitor (Roche, complete, EDTA-free, cat. 11697498001) using a needle (23G, 0.34 mm) and syringe. The yield of crude fraction protein was in the 10–20 μg μL^-1^ range measured by the Thermo Scientific PierceProtein Assay Kit (cat. 23227). The microsomal membrane fraction was stored at –80°C prior to assay.

### ASSAY OF UDP-GLUCURONIC ACID DECARBOXYLASE ACTIVITY

The activity of UXS was performed in a standard reaction mixture (50 μL) at 25°C for 30 min containing 80 mM Tris ⋅ HCl (pH 7.4), 1 mM UDP-GlcA, 1 mM NAD^+^, and 4 μg μL^-1^ microsomal fraction protein (Bar-Peled et al., 2001). Reactions were stopped by the addition of 50 μL of phenol/chloroform (1:1, v/v), vortex-mixed, and subjected to centrifugation (Eppendorf, 10,000 *g*, 5 min, room temperature). The upper phase was retained, and the lower phase was re-extracted with 100 μL ddH_2_O. The two aqueous phases were pooled and analyzed by high-performance liquid chromatography (HPLC) using a Cosmosil C18-AR-II column (250 mm × 4.6 mm; Nacalai Tesque, Kyoto, Japan) run at 1 mL min^-1^ and monitored for UV absorbance (Agilent 1100 HPLC systems, Sig_260nm_, Ref_360nm_). Buffer A was 20 mM triethylamine-acetate (pH 7) and buffer B was 20 mM triethylamine-acetate (pH 7) containing 10% acetonitrile. The gradient program was: 0 to 15 min (0% B), 15.1 to 30 min (15% B) and post-run time 5 min (Rosenberger et al., 2012).

### ^1^H-NMR SPECTROSCOPIC ANALYSIS

UV-absorbing peaks eluted from the Cosmosil column were collected and applied to a Sep-Pak C18 short cartridge according to the procedure described above. The solution was concentrated to dryness under a flow of nitrogen and the dry powder dissolved in D_2_O in 3-mm standard nuclear magnetic resonance (NMR) tubes. NMR spectroscopy was performed on a Bruker AV600 spectrometer. ^1^H chemical shifts were referenced to sodium 2,2-dimethyl-2-silapentane-5-sulfonate.

### ASSAY OF XYLOSYLTRANSFERASE ACTIVITY

The activity of XylT was performed in a standard reaction mixture (90 μL) at 20°C for various times containing 50 mM Hepes-KOH (pH 6.8), 5 mM MnCl_2_, 1 mM DTT, 0.5% Triton X-100, 0.1 mM UDP-Xyl, 0.5 μM Xyl_n_-AA, and 4 μg μL^-1^ microsomal fraction. The reaction mixture was centrifuged at 3,000 *g* for 3 min after termination with 0.3 M acetic acid (5 μL). The supernatant (5 μL) filtered through a membrane filter (pore size 0.22 μm) was subjected to reversed-phase HPLC and monitored for fluorescence (Agilent 1100 HPLC systems, Ex_320nm_, Em_420nm_). The incorporated Xyl_n_-AA products were separated on 2.1 mm × 250 mm, 1.8 μm ZORBAX Eclipse XDB-C_18_ column at a flow rate of 0.5 mL min^-1^ and a column temperature of 20°C. Buffer A was 50 mM sodium acetate buffer (pH 4.3) and buffer B was acetonitrile. The gradient program was: 0 to 5 min (8% B), 5.1 to 25 min (20% B), 25.1 to 30 min (40% B), 30.1 to 35 min (100% B), 35 to 42 min (0% B), and post-run time 5 min.

### MATRIX-ASSISTED LASER DESORPTION IONIZATION-TIME-OF-FLIGHT-MASS SPECTROMETRY

The products of XylT reactions with Xyl_5_-AA as acceptor were analyzed by matrix-assisted laser desorption ionization-time-of-flight-mass spectrometry (MALDI-TOF-MS; Bruker Mircoflex^7^). The enzyme reaction mixture was purified with Sep-Pak C18 short cartridge (Waters) that had been pre-washed with 60% acetonitrile in 0.1% formic acid. The cartridge was washed with 0.1% formic acid (10 mL) and then with 60% acetonitrile in 0.1% formic acid (2 mL) to elute Xyl_n_-AA. The solution was concentrated to dryness under a flow of nitrogen gas. The dry powder was dissolved in water and mixed with the MALDI matrix (0.2 M 2,5-dihydroxybenzoic acid and 0.06 M 1-hydroxyisoquinoline in 50% acetonitrile). The mixture was dried on the stainless steel target plate and detected with MALDI-TOF-MS.

### TREATMENT OF XYLO-OLIGOSACCHARIDES WITH ENDO-β-XYLANASE AND EXO-β-XYLOSIDASE

The degradation of xylo-oligosaccharides was performed according to the method previously described by Lee et al. (2007a). Briefly, the XylT reaction using Xyl_n_-AA acceptors as described above were terminated by heating at 100°C for 5 min and then centrifuged at 12,000 ×*g* for 5 min. The supernatant was treated with endo-β-xylanase (9 U, Sigma) and exo-β-xylosidase (0.01 U, Megazyme) for 6 h at 37°C. The reaction was terminated with 0.1 M acetic acid, and subjected to reverse-phase HPLC analysis.

### RNA EXTRACTION, cDNA LIBRARY CONSTRUCTION, AND RNA-SEQ

Total RNA of apical, middle and basal parts of the stem were extracted with a plant total RNA kit (OMEGA^8^) from 5 plants (tissue cultural plant) with the similar height of 1.2 m. Both the quantity and quality of the RNA were verified with NanoDrop ND 1000 (Thermo Scientific) and the RNA pool was constructed from all five samples with equally 30 μg RNA. The three cDNA libraries were constructed by NEBNext mRNA Library Prep Master Mix Set for Illumina (NEB, E6110) and NEBNext Multiplex Oligos for Illumina (NEB, E7500). Finally, the qualified cDNA libraries detected with Library Quantification Kit-Illumina GA Universal (Kapa, KK4824) were sequenced using IlluminaHiSeq^TM^ 2000. The raw reads were first filtered by removing the adapter sequences, low quality sequences and reads with unknown bases. The sum of the reads from the three samples was obtained to get a whole transcriptome reference profile.

*De novo* assembly was carried out using the Trinity assembly program (Grabherr et al., 2011). Short reads were first assembled into longer but gapless contigs and then the reads were mapped back to contigs to construct scaffolds using the paired-end information. Scaffolds were generated by Trinity software,. Trinity connected the contigs using N to represent unknown sequences between each pair of contigs. The gaps of scaffolds were filled by pure reads in order to get the sequence with the least N bases, and through this process, the unigene sequences were generated. Pure reads of the three samples were mapped to the transcriptome reference unigenes using Bowtie software (Langmead et al., 2009). The number of unambiguous pure reads for each unigene was counted and then normalized into an RPKM value (reads per kilobase per million pure reads). All the sequences of unigenes have been deposited in the Transcriptome Shotgun Assembly Sequence Database (TSA) at NCBI with accession number from GASC01000001 to GASC01055370.

### RNA EXTRACTION, cDNA SYNTHESIS, AND qRT-PCR ANALYSIS

The unigenes related to heteroxylan synthesis were selected for validation by quantitative real-time PCR (qRT-PCR). Total RNA used for qRT-PCR analysis was extracted from the three parts of stem (apical, middle, and basal) according to the procedures described above. The first-strand cDNA was synthesized from 4.0 μg DNA-free RNA using PrimeScript II first Strand cDNA Synthesis Kit (Takara^9^) according to the manufacture’s protocol and used as template at a 1:10 dilution for qRT-PCR. Primers for q-RCR analysis were designed according to the unigenes sequence (**Table S1**). The qRT-PCR was performed according to the protocol of SYBR Premix Ex Taq^TM^ II (Takara) in a 20 μL volume with the Roche LightCyler 480 system (Roche^10^). Cyclophilin was used as the reference gene for all target genes examined.

## RESULTS

### THE SECONDARY THICKNESS OF XYLARY FIBERS VARIES IN DIFFERENT STEM SEGMENTS

**FIGURE 1:**
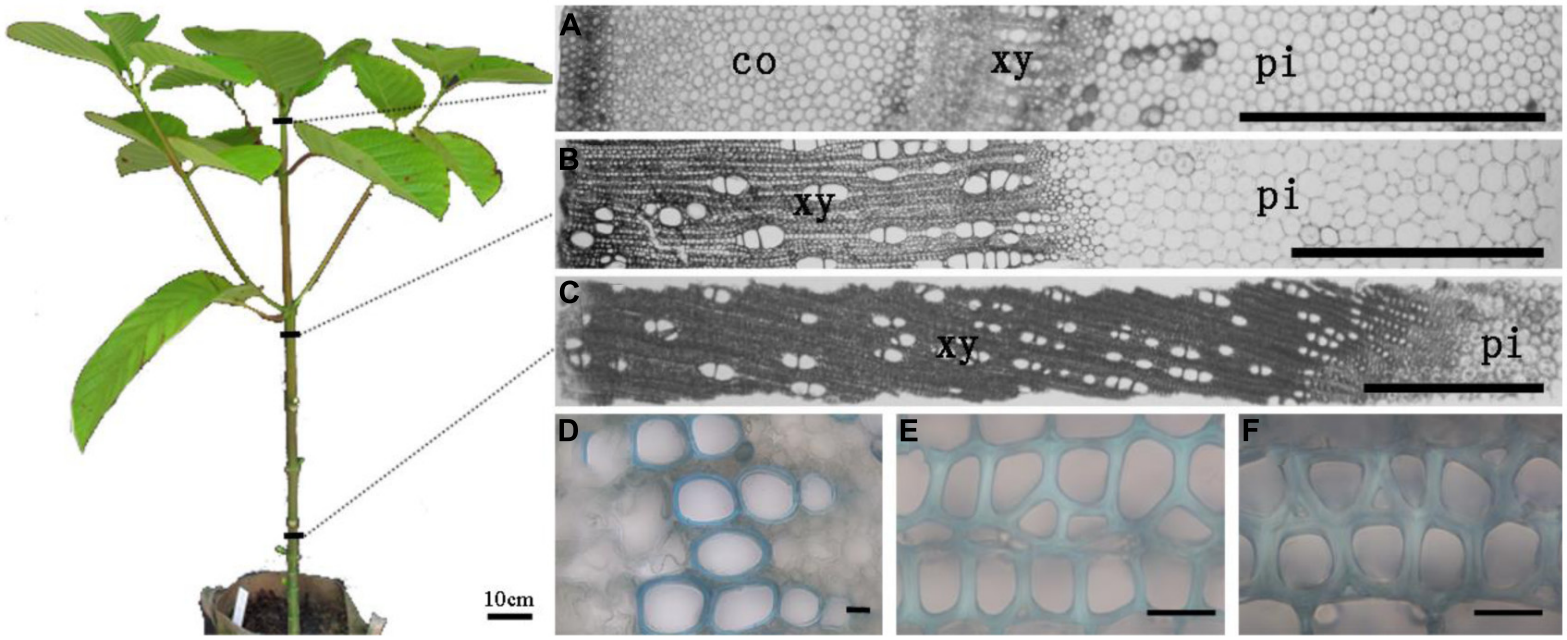
**The sections of apical, middle, and basal segments of stem (A–C) and the secondary wall thickness in xylem (D–F), respectively. (A)** Section of the apical part of the stem with cortex; **(B,C)** Section of middle and basal parts of stem without cortex have different thickness of xylem. **(D–F)** The sections were stained in 0.02% (w/v) toluidine blue O. Only vessel elements have secondary thickening in the top part of stem. The thickness of xylem fiber wall in the middle and bottom parts shows distinct variation. Scale bar: **(A–C)** = 1 mm and **(D–F)** = 20 μm. co, cortex; xy, xylem; pi, pith.

To systematically study heteroxylan synthesis occurring during secondary growth, three different stem segments representing discrete developmental stages with regard to secondary growth were isolated from the apical, middle, and basal regions of 1-year-old *N. cadamba* stems (**Figure 1**). The first segment (referred to as the apical segment) was below the apical meristem, where vascular bundles are formed from procambial cells and consist of primary xylem tissues (**Figures 1A,D**). The second segment (referred to as the middle segment) was from the central region of the stem, where secondary thickening has started (**Figures 1B,E**). The third segment (referred to as the basal segment) was from the basal region of the stem, where secondary xylem fibers are already heavily lignified and the amount of secondary xylem has increased (**Figures 1C,F**). The xylem diameters in the middle and basal stem segments were 1.9 and 4.8 mm, respectively, and displayed obvious secondary thickening of the xylem fiber walls (**Figures 1E,F**). The xylem fiber cell wall thickness was 3.85 μm in the middle segment and 6.17 μm in the basal stem segment.

**FIGURE 2:**
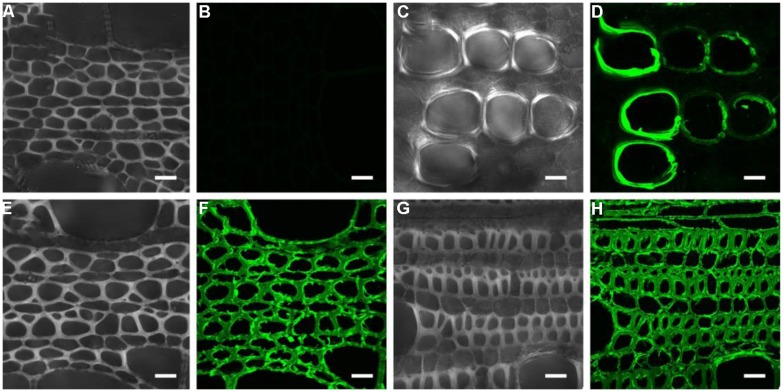
**Immuno-localization of the LM10 epitope in different parts of the stem. (A,B)** middle stem part section without LM10 antibody but with fluorescein isothiocyanate-conjugated secondary antibodies immuno-localization as control; **(C–H)** apical **(C,D)**, middle **(E,F)**, and basal **(G,H)** stem part sections probed with the LM10 antibody then secondary antibody to immuno-localize heteroxylans. Scale bar: 20 μm.

To investigate heteroxylan distribution in the stem, the LM10 monoclonal antibody was used to immuno-localize heteroxylan. LM10 can bind to unsubstituted and relatively low-substituted heteroxylans but not to wheat AX (McCartney et al., 2005). In the apical stem segment, only vessel elements showed LM10 binding. However, strong signals were detected in the xylem of middle and basal stem segments (**Figure 2**). All sections have showed the same appearance and we displayed one section for each stem segment in **Figure 2**.

### NON-CELLULOSIC POLYSACCHARIDE AS WELL AS CELLULOSE AND LIGNIN CONTENT VARIES IN DIFFERENT STEM SEGMENTS

To investigate the distribution of xylogenesis in different regions of the stem, non-cellulosic polysaccharide as well as the cellulose and lignin content was measured (**Tables 1** and **2**). The levels of cellulose decreased with stem maturity being highest in the apical segment and lower in the middle/basal segments. In contrast the levels of both non-cellulosic polysaccharides and lignin increased with stem maturity being highest in the middle/basal segments and lowest in the apical segment. The major non-cellulosic polysaccharide in the stem segments was heteroxylan with substantially lower levels of xyloglucan and heteromannan (**Table 2**). The heteroxylan levels increased substantially with stem maturity (xylogenesis) whereas both xyloglucan and heteromannan decreased as would be expected in tissues transitioning between primary and secondary wall development (Pauly et al., 2013). The results confirmed that heteroxylan was the major non-cellulosic polysaccharide in stem walls of *N. cadamba*.

**Table 1:** Cellulose, non-cellulosic polysaccharide, and lignin contents (%, w/w) in apical, middle, and basal stem segments.

Sample	Cellulose	Non-cellulosic polysaccharides	Lignin
Apical	53.78 ± 1.27^a^	19.85 ± 2.95^b^	13.64 ± 2.49^b^
Middle	42.80 ± 1.69^b^	25.93 ± 1.01^a^	23.36 ± 1.13^a^
Basal	44.16 ± 0.94^b^	26.86 ± 2.86^a^	23.9 ± 3.59^a^

The data represent means ± SE (*n* = 3). Different letters represent significant differences (*P* < 0.05; one-way ANOVA; Bonferroni *post hoc*).

**Table 2:** Glycosyl linkage composition (mol %) in apical, middle, and basal segments.

Monosaccharide linkage	Apical	Middle	Basal
1,4-Glc	62.6	55.8	54.6
**Total cellulose**	**62.6**	**55.8**	**54.6**
1,4-Xyl (p)	20.45	18.3	25
1,2,4-Xyl (p)	1.05	1.85	2.15
1,3,4-Xyl (p)	0.35	0.9	0.95
1,2,3,4-Xyl (p)	1.95	5.85	4.85
t-Ara	1.3	0.5	0.3
**Total heteroxylan**	**20.45**	**32.65**	**34.95**
1,4-Man (p)	2.05	1.9	1.7
1,4,6-Man (p)	0.3	0.05	0.05
1,4-Glc (p)	2.4	1.7	1.55
1,4,6-Glc (p)	0.3	0.05	0.05
t-Gal	0.6	0.1	0.15
**Total heteromannan**	**5.85**	**3.6**	**3.45**
1,4,6-Glc (p)	1.65	1.25	1.1
1,4-Glc (p)	1.6	1.25	1.15
1,2-Xyl (p)	0.25	0	0
t-Xyl	1.3	0.6	0.5
**Total xyloglucan**	**4.7**	**3**	**2.75**
1,5-Ara (f)	0.75	0.25	0.15
**Total arabinan**	**0.75**	**0.25**	**0.15**
1,3-Glc (p)	0.25	0	0
t-Glc (p)	0.5	0	0
**Total callose**	**0.75**	**0**	**0**

**Total**	**96.4**	**95.7**	**96.2**

Each data represents the mean of double measurements.

### ANALYSIS OF NON-CELLULOSIC POLYSACCHARIDE, CELLULOSE, AND LIGNIN CONTENTS BY FOURIER TRANSFORM INFRARED SPECTROSCOPY

**FIGURE 3:**
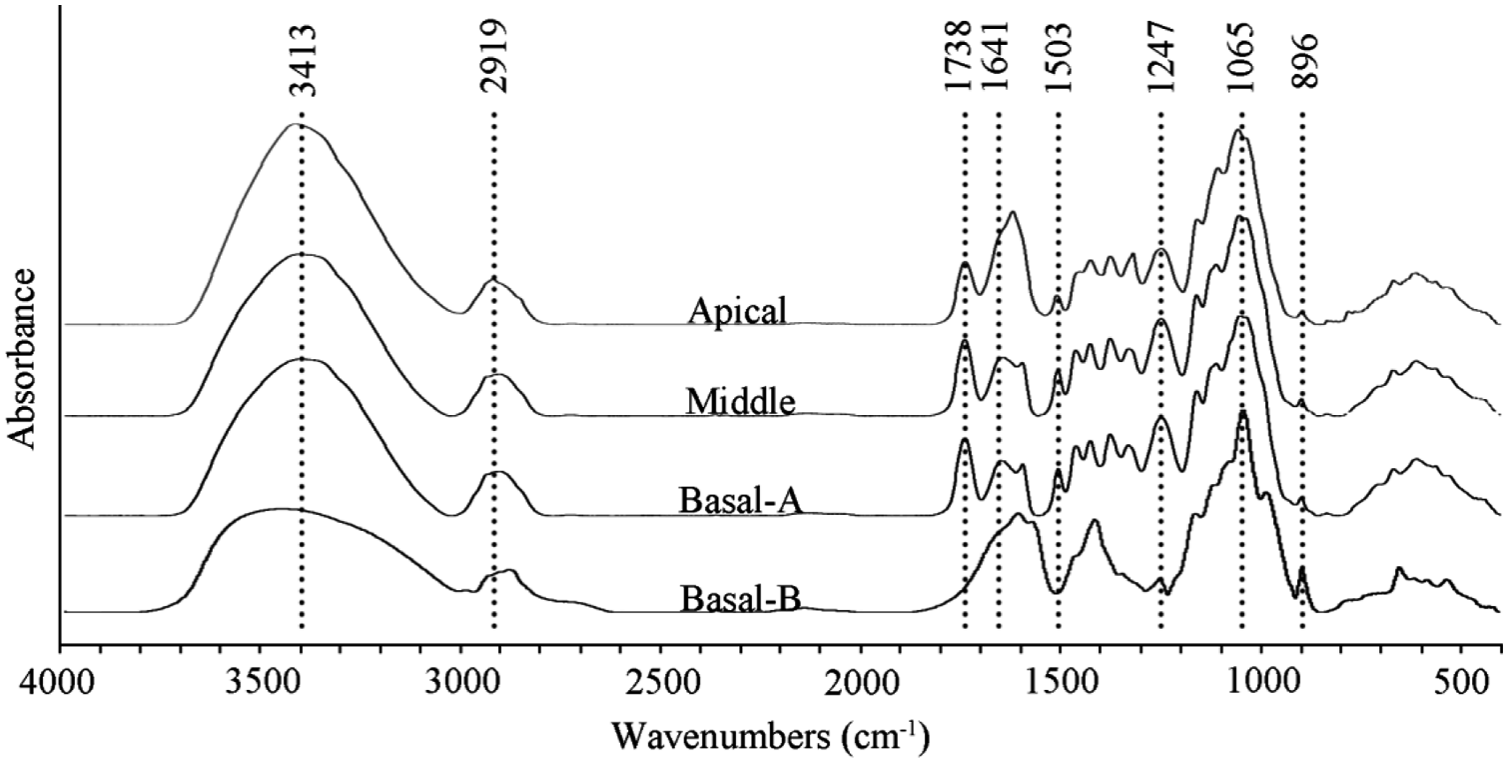
**FT-IR spectra of wood flour [apical, middle, and basal-(A)] and non-cellulosic polysaccharides [basal-(B)] extracted from the basal segment of the stem with 2 N KOH**.

To verify the differences in cell wall composition within the different stem segments, FT-IR was used to detect a profile for each domain (**Figure 3**). The absorption peaks in FT-IR spectra have previously been assigned to functional groups of cell wall components (Schwanninger et al., 2004; Saulnier et al., 2013; Liu et al., 2014). The broad peak around 3413 cm^-1^ corresponds to the O-H hydroxyl groups found in both cellulose and non-cellulosic polysaccharides. The peak around 2919 cm^-1^ is due to the C-H stretch in methyl and methylene groups including symmetric and asymmetric mode stretching. The peaks in the fingerprint region of FT-IR spectra are assigned as follows: 1738 cm^-1^ for the C = O stretching vibration of carboxyl, carbonyl and acetyl groups in non-cellulosic polysaccharides, 1641 cm^-1^ for the absorbed water, 1503 cm^-1^ for the C = C stretching of aromatic skeletal in lignin, 1247 cm^-1^ for the C-O stretching in lignin, the bands between 1200 and 1000 cm^-1^ are typical of MGX (Samanta et al., 2012), the large peak 1065 cm^-1^ for C-O, C-C stretching or C-OH bending in non-cellulosic polysaccharides, 896 cm^-1^ for C-H deformation in β-linked glucosidic bond (Kacurakova et al., 2002).

Fourier transform infrared spectroscopy spectra of *N. cadamba* wood powder generated from the three stem sections as well as purified non-cellulosic polysaccharides of the basal stem segment are shown in **Figure 3**. A comparison of the wood powder samples reveals that the 3413 cm^-1^ signal is higher in the apical segment compared to the middle and basal segment. In addition, the peak between 1641 and 1619 cm^-1^ is higher and the 896 cm^-1^ peak is weaker in the apical segment compared to the middle and basal segments, consistent with a higher primary wall content in the apical region, and a lower content of non-cellulosic polysaccharides (**Table 1**).

The extracted non-cellulosic polysaccharides have different FT-IR absorption peaks compared to the total wood powder (**Figure 3**). The decrease of the peak around 3413 cm^-1^ is likely to be due to a reduced O-H signal. The peaks at 1503 and 1247 cm^-1^, assigned to lignin, are virtually absent and the loss of the peak at 1738 cm^-1^ in the non-cellulosic polysaccharides sample is due to saponification of the acetyl ester bond. There are few differences between the extracted non-cellulosic polysaccharides and wood powder in the 1200–1000 cm^-1^ region of the spectrum which indicates that heteroxylan is the main non-cellulosic polysaccharides in *N. cadamba.* The sharp band at 896 cm^-1^ is attributed to β-glycosidic linkages between the sugars units, indicating that the residues forming the backbone of the polysaccharides are linked by covalent bonds in the β-configuration.

### UDP-XYL SYNTHASE ACTIVITY IN MICROSOMAL FRACTION

**FIGURE 4:**
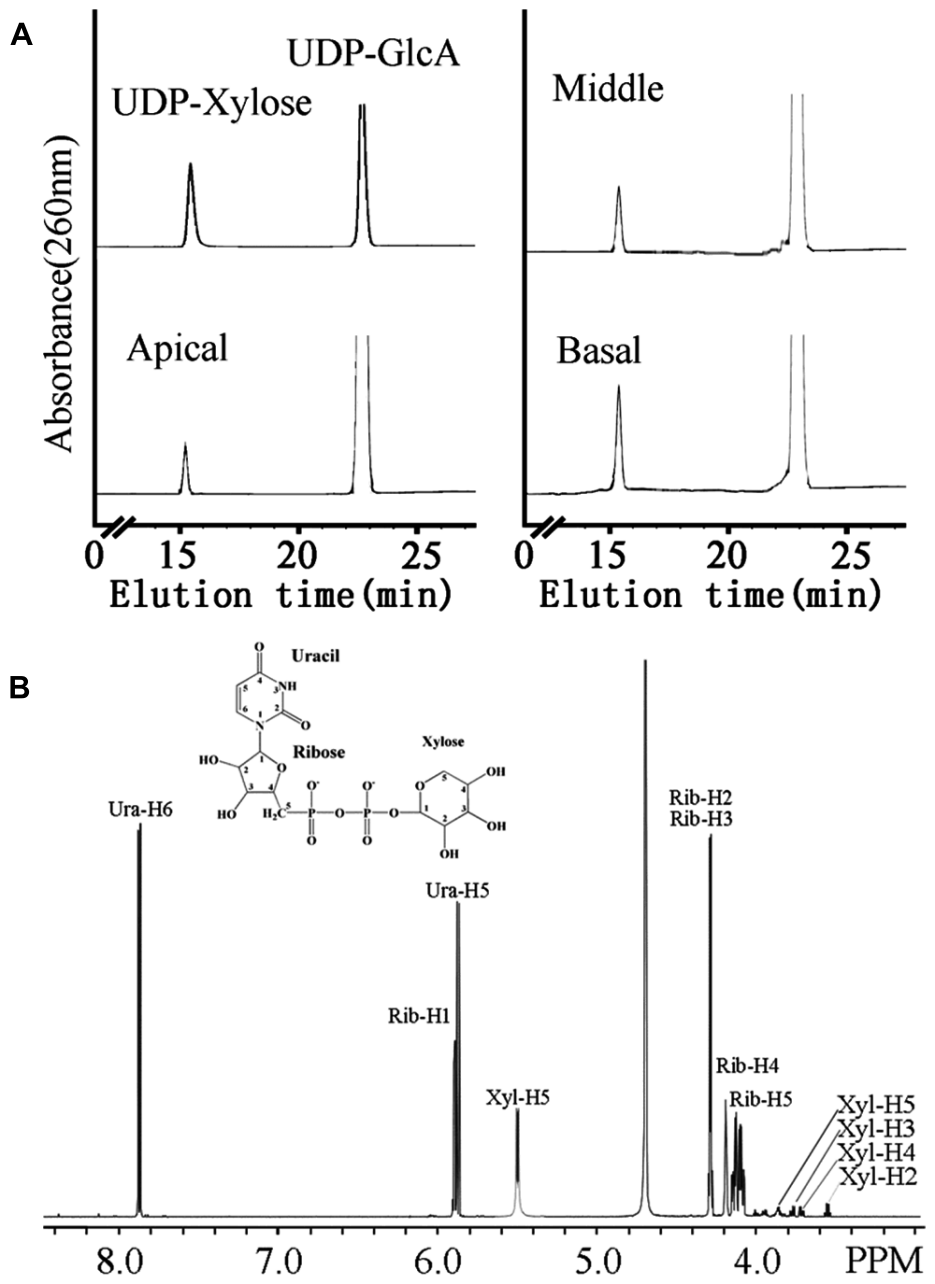
**The UXS activity of microsomes from apical, middle and basal regions (A) and one-dimensional proton NMR spectrum of the product of the UXS assay (B). (A)** Microsomes were incubated with 1 mM UDP-GlcA and 1 mM NAD^+^. The products of the reaction were separated on a Cosmosil C18-AR-II column. The retention times of standard UDP-Xyl and UDP-GlcA are indicated. **(B)** Chemical shifts were referenced to sodium 2,2-dimethyl-2-silapentane-5-sulfonate. The structure of UDP-Xyl is shown.

UDP-Xyl is the activated sugar substrate utilized in heteroxylan synthesis. In plants, the biosynthesis of UDP-Xyl from UDP-glucuronic acid (UDP-GlcA) is catalyzed by numerous UDP-GlcA decarboxylase (also referred to as UXS) isoforms. UXS activity was tested in microsomal membrane preparations in a reaction converting UDP-GlcA to UDP-Xyl. Microsomal membranes from the middle (7.01 mmol UDP-Xyl min^-1^ mg^-1^) and basal (4.87 mmol UDP-Xyl min^-1^ mg^-1^) stem segments had higher UXS activity than those from the apical (2.39 mmol UDP-Xyl min^-1^ mg^-1^) region (**Figure 4A**). To confirm the product of the reaction, NMR was used to decode the product of UDP-Xyl peak. The one-dimensional proton spectrum of the reaction products (**Figure 4B**) matched the UDP-Xyl standard and the spectrum of previous studies (Bar-Peled et al., 2001; Gu et al., 2011), confirming that the product of the UXS activity assay was indeed UDP-Xyl.

### XYLOSYLTRANSFERASE ACTIVITY IN MICROSOMAL MEMBRANES EXTRACTED FROM DIFFERENT STEM SEGMENTS

**FIGURE 5:**
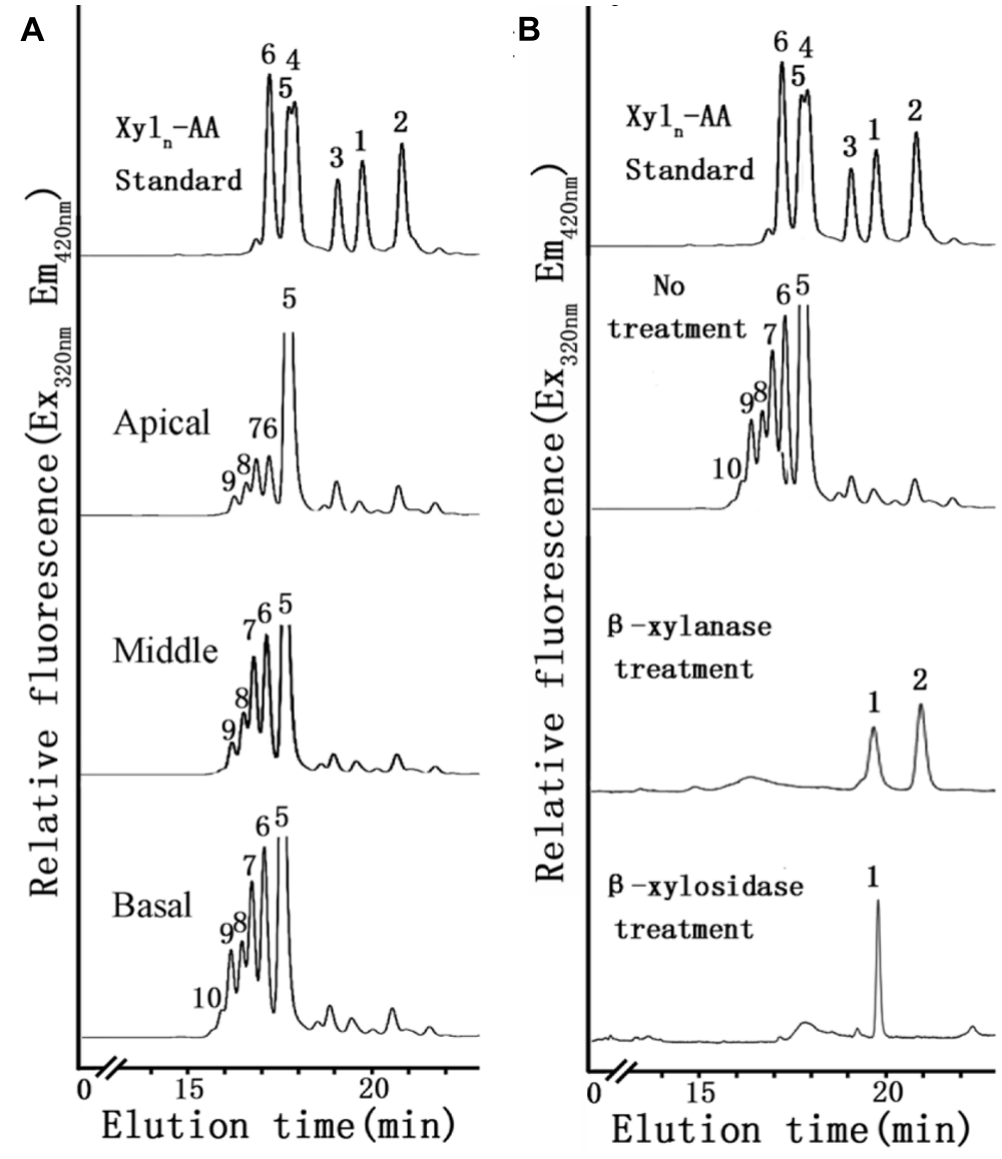
**XylT activity of microsomes using Xyl_5_-AA as an acceptor from apical, middle and basal **(A)** and degradation of the XylT-catalyzed reaction products by an endo-β-xylanase and an exo-β-xylosidase (B). (A)** Microsomes were incubated with UDP-Xyl and the fluorescent acceptor Xyl_5_-AA for 6 h. The reaction products were subjected to reversed-phase HPLC for separation and detection of the fluorescent xylo-oligosaccharides. The retention times of the standards Xyl_1_-AA to Xyl_6_-AA are indicated. The numbers above the peaks indicate the DP of the oligosaccharides. **(B)** The products Xyl_1_–Xyl_2_ of degradation indicated that the reaction products have a β-(1,4)-linkage.

To identify differences in heteroxylan biosynthesis during xylogenesis, XylT activity was measured in microsomes extracted from the different stem segments. A xylo-oligosaccharide fluorescently tagged at its reducing end with anthranilic acid (Xyl_5_-AA) was used as the acceptor in the XylT activity assay. Microsomal membranes from the apical region of the stem exhibited the lowest XylT activity whereas those from the basal segment had the highest XylT activity, as evidenced by the greater number of Xyl additions to the acceptor and the increased relative peak area of each xylo-oligosaccharide (Xyl_6_–Xyl_10_; **Figure 5A**).

**FIGURE 6:**
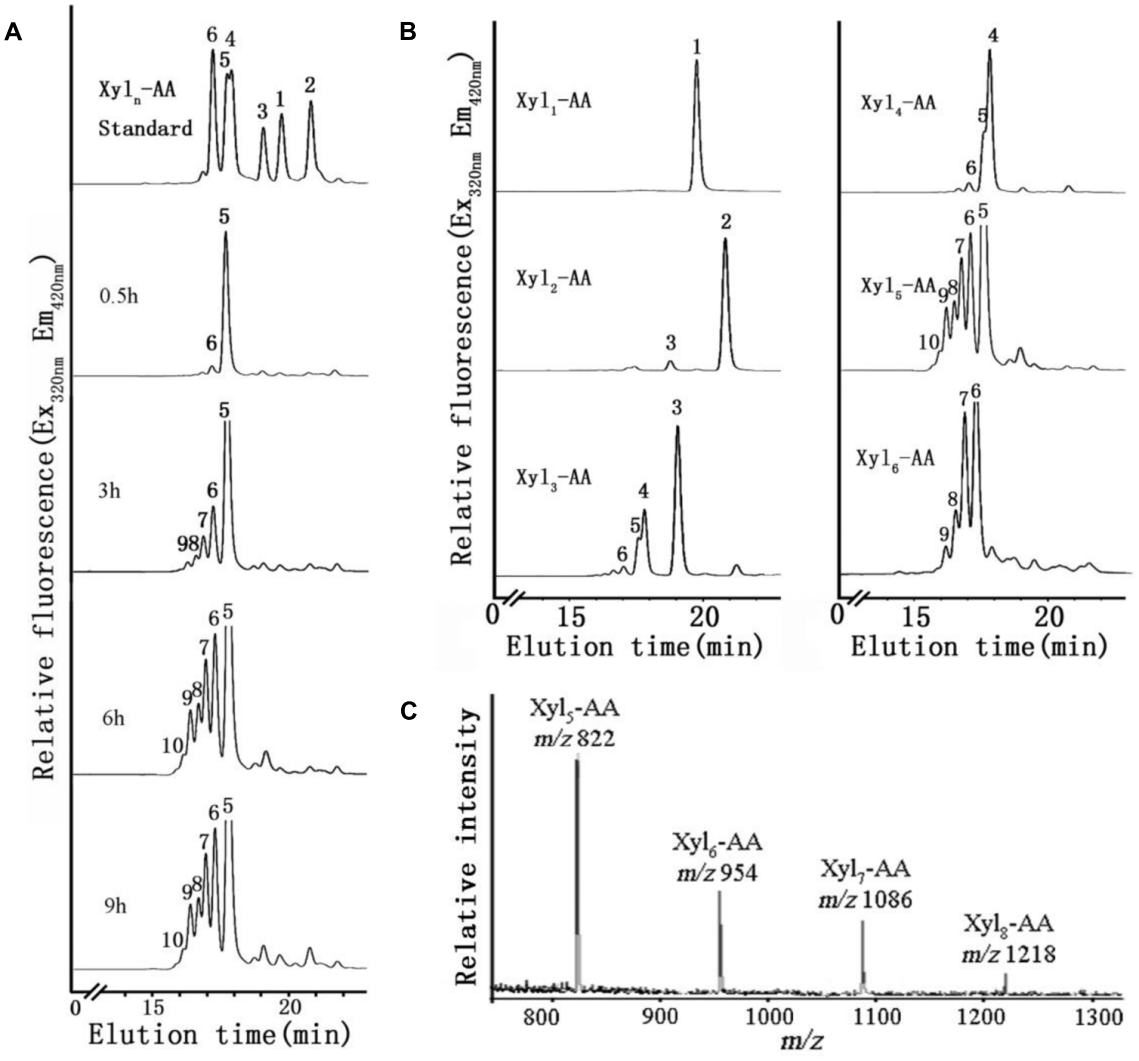
**XylT activity of microsome from the basal region and MALDI-TOF MS spectrum of the purified reaction products. (A)** Microsomes were incubated with UDP-Xyl and the fluorescent acceptor Xyl_5_-AA for 0.5, 3, 6, and 9 h. **(B)** Microsomes were incubated with UDP-Xyl and the fluorescent acceptors Xyl_1_-AA, Xyl_2_-AA, Xyl_3_-AA, Xyl_4_-AA, Xyl_5_-AA, and Xyl_6_-AA for 6 h. **(C)** MALDI-TOF MS spectrum of the purified reaction products produced by microsomes isolated from the basal stem segment using Xyl_5_-AA as an acceptor. In the spectrum, a mass increment of 132 Da was observed matching a pentosyl residue. The series of ions matched the products Xyl_5_-AA to Xyl_8_-AA.

A time-course of XylT activity was conducted over a 9 h period using microsomal membranes from the basal region of the stem (**Figure 6A**). Only a small amount of Xyl_6_ was generated after 0.5 h incubation. After 3 h, an increased amount of Xyl_6_ was generated and longer (Xyl_7_–Xyl_9_) xylo-ologosaccharides started to appear. After a 6 h incubation, up to five xylosyl residues were added to Xyl_5_-AA leading to the generation of xylo-oligosaccharides ranging from Xyl_6_ to Xyl_10_ with no discernible difference observed after incubation for 9 h. Therefore, the XylT assay was shown to be time-dependent and 6 h was chosen as the standard time period over which subsequent assays were conducted.

We next investigated the effect of xylo-oligosaccharide length as acceptors for XylT activity of microsomal membranes from the basal stem segment (**Figure 6B**). No XylT activity was detected using the monomer (Xyl_1_-AA) and only a low activity was observed with the dimer (Xyl_2_-AA), similar to results found in *Arabidopsis* (Lee et al., 2007a). Higher XylT activity was detected using Xyl_3_-AA to Xyl_6_-AA as acceptors, with Xyl_5_-AA being the best acceptor.

To verify that Xyl addition occurred at the O-4 position and was in the β-configuration, the reaction products generated by microsomal membranes from the basal stem segment using Xyl_5_-AA and UDP-Xyl were analyzed by MALDI-TOF-MS and enzymatic treatment. MALDI-TOF-MS identified products with masses differing by 132 Da (*m/z* 822, 954, 1086, 1218), consistent with the addition of Xyl residues to Xyl_5_-AA (**Figure 6C**). When the XylT-generated products were treated with an endo-β-xylanase or exo-β-xylosidase, the products were hydrolyzed to monomeric or dimeric xylo-oligosaccharides (**Figure 5B**). These results confirmed that the products were composed of 1,4-β-D-Xyl-linked residues.

### TRANSCRIPT PROFILING OF DIFFERENT PART OF STEMS

**FIGURE 7:**
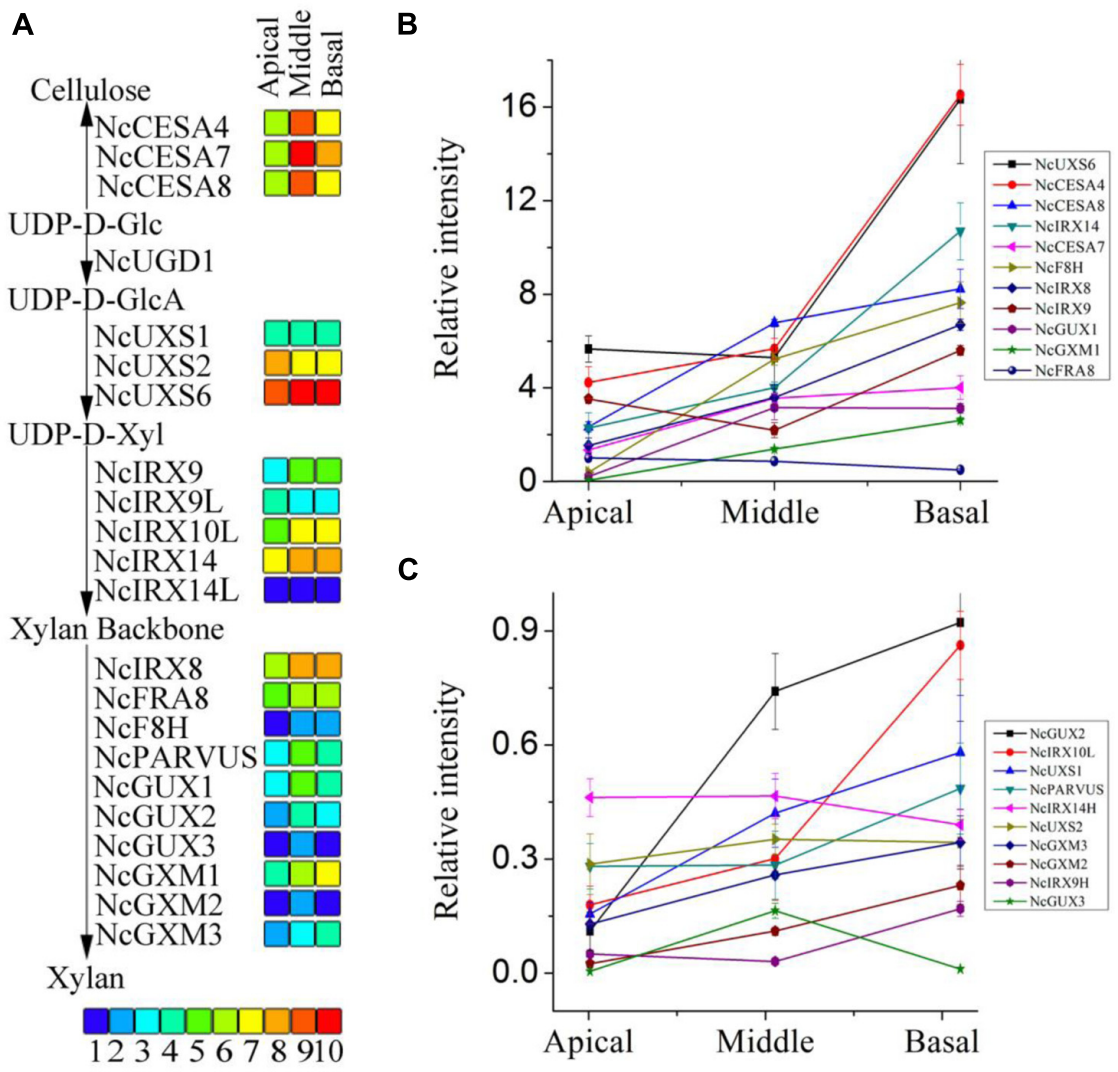
**RPKM values and expression of the unigenes related to heteroxylan synthesis. (A)** The RPKM values of each unigene are shown by three grids, with the left to the right representing the apical, middle, and basal regions, respectively. The grids with 10 different colors from blue to red show the RPKM values 0–10, 10–20, 20–40, 40–60, 60–100, 100–140, 140–250, 250–330, 330–500, and over 500 represented by colors 1–10, respectively. **(B,C)** qRT-PCR was performed to the unigenes related to heteroxylan synthesis. Each data point represents the means ± SD (*n* = 3).

Transcript profiling was used in order to identify the genetic basis for differences in heteroxylan synthesis during xylogenesis in the stem of *N. cadamba*. Three libraries were generated from stem material from the apical, middle, and basal segments and used for RNA-seq analysis to obtain an overview of the *N. cadamba* transcriptome. The mixed reads from the three libraries were used to construct the whole transcriptome pool, and were also used as the reference and in combination with data from each separate sample to perform further gene expression analysis. RNA-seq analysis of *N. cadamba* stem in total, more than 111.64 million reads were generated. A *de novo* assembly produced a transcriptome containing 111,864 contigs and 55,432 unigenes (**Table 3**). The unigenes were annotated by NCBI non-redundant (NR), Swiss-Prot, Gene Ontology (GO), Clusters of Orthologous Groups (COGs), and Kyoto Encylopedia of Genes and Genomes (KEGGs) databases. The results indicated that of 55,432 unigenes, 26,404 (47.63%) had a significant similarity to known proteins in the NR database. Some heteroxylan synthesis relative candidate genes have been found in *N. cadamba* (**Figure 7A**).

**Table 3:** Throughput and quality of RNA-seq of *N. Cadamba.*

Libraries	Raw reads	Total nucleotides (nt)	GC (%)	Q20%	Q30%
Apical	25,085,358	5,066,725,234	46.03	88.95	81.51
Middle	38,555,036	7,787,264,195	44.64	88.98	82.13
Basal	47,999,394	9,695,200,117	44.29	89.41	82.53

### CANDIDATE GENES RELATED TO HETEROXYLAN SYNTHESIS

The three stem segments used for the RNA-seq analysis have different degrees of secondary cell wall thickening. Genes encoding the secondary cell wall-related cellulose synthase (CESA) catalytic subunits, *CESA4*, *CESA7*, and *CESA8,* are regarded as reference genes to identify genes that are co-expressed during secondary cell wall synthesis (Brown et al., 2005; Persson et al., 2005). Estimation of gene expression (RPKM, Reads Per Kilobase Per Million) values of the three likely orthologous *NcCESA* genes show that the middle part has the highest expression and that the apical segment the lowest (**Figure 7A**), correlating with xylogenesis and indicating that the RNA-seq data can be mined for secondary wall polysaccharide synthesis genes. The other CESA transcripts show the highest expression at the apical segment and decreasing with xylogenesis (**Figure S1**), suggesting that they are involved in the synthesis of cellulose in the primary cell wall. The results are consistent with the expression pattern of the corresponding *Arabidopsis* genes, and on the other hand support the results why apical segment have relative high cellulose component.

Heteroxylan is the major non-cellulosic polysaccharide in the secondary walls of dicot plants. Putative orthologs of genes encod-ing enzymes involved in heteroxylan synthesis in *Arabidopsis* were discovered and selected to provide an overview of heteroxylan syn-thesis in *N. cadamba* (**Figure S2**). The transcripts of close homologs of *UXS*, which played important role in the substrate of the xylan backbone formation (Harper and Bar-Peled, 2002; Pattathil et al., 2005), were identified abundant with xylogenesis of stem (**Figure 7A**). This is keeping with our UXS activity from stem protein extraction. These genes included those encoding enzymes *IRX9*, *IRX10*, and *IRX14* involved in xylan backbone elongation in *Arabidopsis* (Wu et al., 2009, 2010; Lee et al., 2010) and homologs also showed abundant in the middle and base stem, except *NcIRX9L* (**Figure 7A**). Consistent with xylan backbone, the transcripts of genes *IRX8, FRA8, PARVUS* involved in reducing end tetrasaccharide synthesis were also highly expressed in the middle and basal stem segments compared to the apical segment (**Figure 7A**). Additionally, genes recently characterized in xylan side chain GlcA methylation (GUX; Mortimer et al., 2010; Rennie et al., 2012; Bromley et al., 2013) and genes characterized in xylan *O*-acetylation (GXM; Lee et al., 2012a; Urbanowicz et al., 2012) were also mostly increasing in the middle and basal stem segments (**Figure 7A**). The annotations of these genes are shown in **Tables S2–S4**. Higher xylan synthesis genes expressed in the middle and basal stem segments (**Figure 7A**), is consistent with the higher heteroxylan synthesis observed in these two regions of the stem (**Figure 5A**). To examine transcripts result, the qRT-PCR was used to confirm the results and conclude that almost all of these genes had higher expression levels in the middle and basal segments (**Figures 7B,C**). These results indicate that heteroxylan synthesis is active with the xylogenesis in *N. cadamba*.

## DISCUSSION

### HETEROXYLAN IS THE PREDOMINANT NON-CELLULOSIC POLYSACCHARIDE IN *N. Cadamba* STEMS

Cellulose, non-cellulosic polysaccharides and lignin are the main components of wood, grass straw, agricultural residues, forestry, and municipal solid wastes (Perez et al., 2002). The composition of these materials varies depending on the plant species (Pauly and Keegstra, 2008). The secondary wall includes two main kinds of non-cellulosic polysaccharides: heteromannans and heteroxylans (Pauly et al., 2013). Heteromannans are found mainly in the gymnosperms whereas heteroxylans are mainly found in the dicots and all types of walls in commelinoid monocots (Pauly and Keegstra, 2008). Data from this study shows that heteroxylan comprises a significant proportion of the walls of *N. cadamba* stems (**Table 2**). In addition, application of the LM10 antibody which is specific for heteroxylans generated a strong signal in xylem (**Figure 2**), demonstrating that heteroxylan is the dominant non-cellulosic polysaccharide in *N. cadamba.*

### *N. cadamba* HAS A HIGH XylT ACTIVITY

Xylosyltransferase activities capable of producing xylo-oligosaccharides in microsomal membranes have been identified in various plants, such as wheat seedlings, barley endosperm and *Arabidopsis* (Kuroyama and Tsumuraya, 2001; Urahara et al., 2004; Lee et al., 2007a). In the present study, it has been shown that the XylT activity from *N. cadamba* microsomal membranes can transfer up to five Xyl residues onto a Xyl_5_ acceptor (**Figures 5** and **6**) and that xylo-oligosaccharides as short as xylobiose and xylotriose can act as acceptors (**Figure 6B**). However, the measured XylT activity of *N. cadamba* is lower compared to *Arabidopsis* (Lee et al., 2012b) and poplar (Lee et al., 2012c). UXS activity, which catalyzes heteroxylan donor substrate UDP-Xyl formation, was also detected in the *N. cadamba* microsomal membranes (**Figure 4**). Although there are six and seven *UXS* gene members in *Arabidopsis* and poplar, respectively (Harper and Bar-Peled, 2002; Du et al., 2013), only some of the encoded UXS proteins are predicted to have an NH_2_-terminal transmembrane domain with AtUXS1, AtUXS2, and AtUXS4 confirmed to locate to the Golgi rather than the cytosol. It is therefore possible that the other UXS enzymes function in another sub-cellular location and hence the USX activity assay in *N. cadamba* microsomes may only represent a proportion of the total cellular UXS activity. Taken together, our enzymatic assay results show that *N. cadamba* is a good model to analyze the heteroxylan synthesis pathway during xylogenesis.

Previous research has demonstrated that the deposition of heteroxylan is associated with the development of tissues with a high content of secondary walls with vascular and supporting structures, which may eventually be lignified (Waldron and Selvendran, 1992; Femenia et al., 1999). Results from this study indicate that the highest XylT activity is associated with the basal stem segment of *N. cadamba,* with the apical segment showing the lowest activity, thus correlating XylT with secondary thickening in the middle and basal regions of the stem (**Figures 1E,F**).

### HETEROXYLAN CAN BE REGARDED AS A REFERENCE FOR XYLOGENESIS

Heteroxylan has been proposed to associate with cellulose microfibrils by hydrogen bonding, which is influenced by heteroxylan substitution patterns (Bromley et al., 2013). Heteroxylan may also be covalently linked to lignin via ester bonds to GlcA (Imamura et al., 1994; Balakshin et al., 2011). Analysis of heteroxylan distribution and composition in *N. cadamba* stems (**Tables 1** and **2**; **Figure 2**) provides evidence that increased heteroxylan accompanies wood formation. The apical stem segment has a higher cellulose content than the middle and basal parts which may be due in part to the fact that the apical region of the stem contains a higher proportion of cells undergoing primary wall synthesis which have a lower heteroxylan content.

Fourier transform infrared spectroscopy spectroscopy has been widely used in wood chemistry to characterize wood polymers both qualitatively and quantitatively (Schwanninger et al., 2004; Yang et al., 2007; Saulnier et al., 2013). Our FT-IR data of wood powder showed that the middle and basal regions of the stem have almost the same absorbance curve, indicating the existence of similar functional groups and therefore similar abundances (**Figure 3**). This is consistent with the result of wall component analysis that the middle and basal segments have almost same percentages of cellulose, non-cellulosic polysaccharides and lignin (**Table 1**). The apical stem region also contains the same functional groups as the middle and basal segments but with different relative peak intensities (**Figure 3**). The differences are consistent with the proportion of the three polymers between apical and middle or basal segments. The extracted non-cellulosic polysaccharides from basal stem segment were analyzed using FT-IR (**Figure 3**) with the spectra obtained being consistent with plant tissues rich in heteroxylan such as bamboo stem, wheat straw and *Caragana korshinskii* stalks (Ren et al., 2008; Bian et al., 2010; Wen et al., 2011).

### *N. cadamba* CAN BE USED AS PULPWOOD

Our analysis of the ligno-cellulosic content of the middle stem segment of an 8-year-old *N. cadamba* also showed that cellulose (44.1%), non-cellulosic polysaccharides (30.6%), and lignin (23.3%) content were close to the basal segment of the 1-year-old stem. Compared with other hardwood species, the lignin content of *N. cadamba* is slightly higher than the average level (22.8%), but still lower than *Eucalyptus* (29∼32%; Vassilev et al., 2012). In agro-industrial applications, lignin exerts a negative impact on the utilization of plant biomass in both the biofuel and paper industries. Although *Eucalyptus* is the most important hardwood fiber crops species in tropic and subtropical regions (Villar et al., 2011), *N. cadamba* may be a suitable alternative tree species due to the future demands for biomass utilization.

### GENES RELATED TO HETEROXYLAN SYNTHESIS ARE EXPRESSED DIFFERENTIALLY IN APICAL, MIDDLE, AND BASAL SEGMENTS

High-throughput RNA-seq technology is an effective method to obtain sequence from expressed genes, discover novel genes and investigate gene expression patterns (Varshney et al., 2009). This study applied RNA-seq technology to *N. cadamba*, a species without a reference genome, for characterizing and comparing gene expression profiles between apical, middle, and basal segments of the stem. Previous studies have identified a number of *Arabidopsis* genes that are essential for correct secondary cell wall formation that are co-expressed with CESA genes (Brown et al., 2005; Persson et al., 2005). In *Arabidopsis*, *CESA4, CESA7,* and *CESA8* are required for cellulose synthesis during secondary cell wall formation in vascular tissues. In this study, the three putative *N. cadamba CESA* orthologs have highest expression levels in the middle stem segment and lowest in the apical stem region (**Figure 7A**). For the 18 unigenes presumed to function during heteroxylan synthesis in *N. cadamba* stems, the majority shows a similar expression pattern compared to the *NcCESA* genes (**Figures 7B,C**), indicating our RNA-seq is a useful approach to identify genes that participate in xylogenesis.

## Conflict of Interest Statement

The authors declare that the research was conducted in the absence of any commercial or financial relationships that could be construed as a potential conflict of interest.

## Footnotes

1 http://www.ncbi.nlm.nih.gov/bioproject/PRJNA232616

2 http://www.plantprobes.net/

3 http://www.jacksonimmuno.com

4 http://www.zeiss.com

5 http://www.bruker.com

6 http://www.waringlab.com

7 http://www.bruker.com

8 http://omegabiotek.com

9 http://www.takara-bio.com

10 http://www.roche-applied-science.com

## SUPPLEMENTARY MATERIAL

Table S1 **Primers used in q-RTPCR analysis**.

Table S2 **RPKM quantitation of *N. cadamba* genes profiled by RNA-seq**.

Table S3 **Swissprot annotation of *N. cadamba* genes profiled by RNA-seq**.

Table S4 **NR annotation of *N. cadamba* genes profiled by RNA-seq**.

Figure S1 **Reads per kilobase per million values of other CESA transcripts**.

Figure S2 **Phylogeny tree of genes profiled**.
