# Supplementary figures and images for: Biochemical and molecular changes associated with heteroxylan biosynthesis in Neolamarckia cadamba (Rubiaceae) during xylogenesis
Source: Front Plant Sci. 2014 Nov 7;5:602. doi: 10.3389/fpls.2014.00602 (PMC4224071; doi:10.3389/fpls.2014.00602)

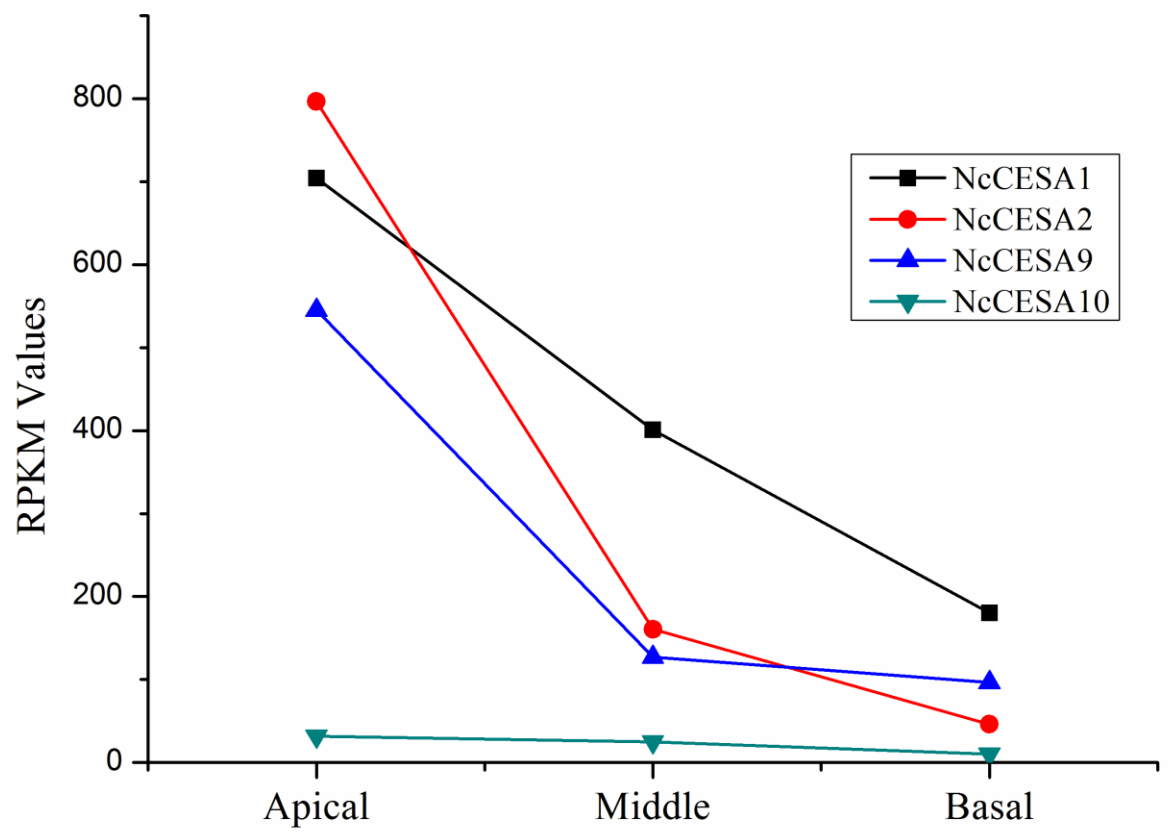

Figure S1. RPKM values of other CESA transcripts.

Supplement: Figure S1 — Reads per kilobase per million values of other CESA transcripts. [file Figure_1.PDF]

A

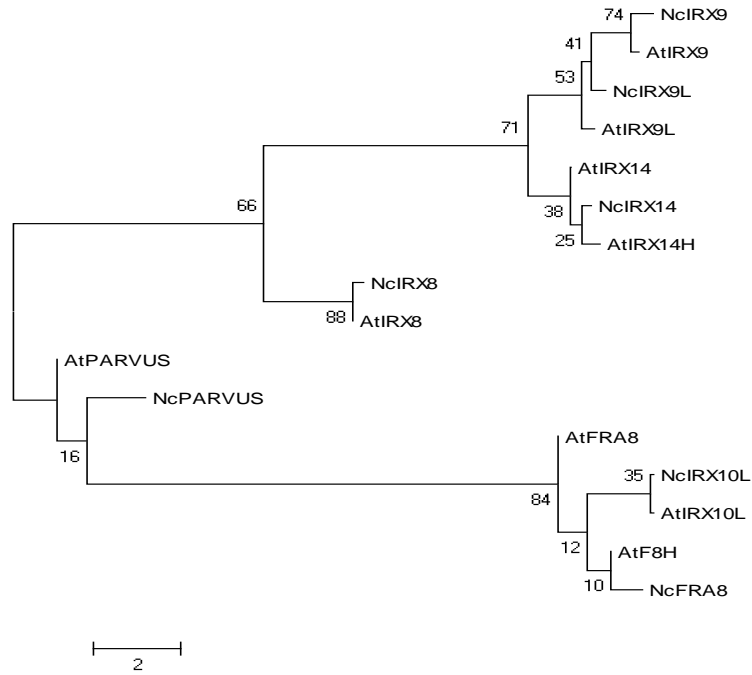

B

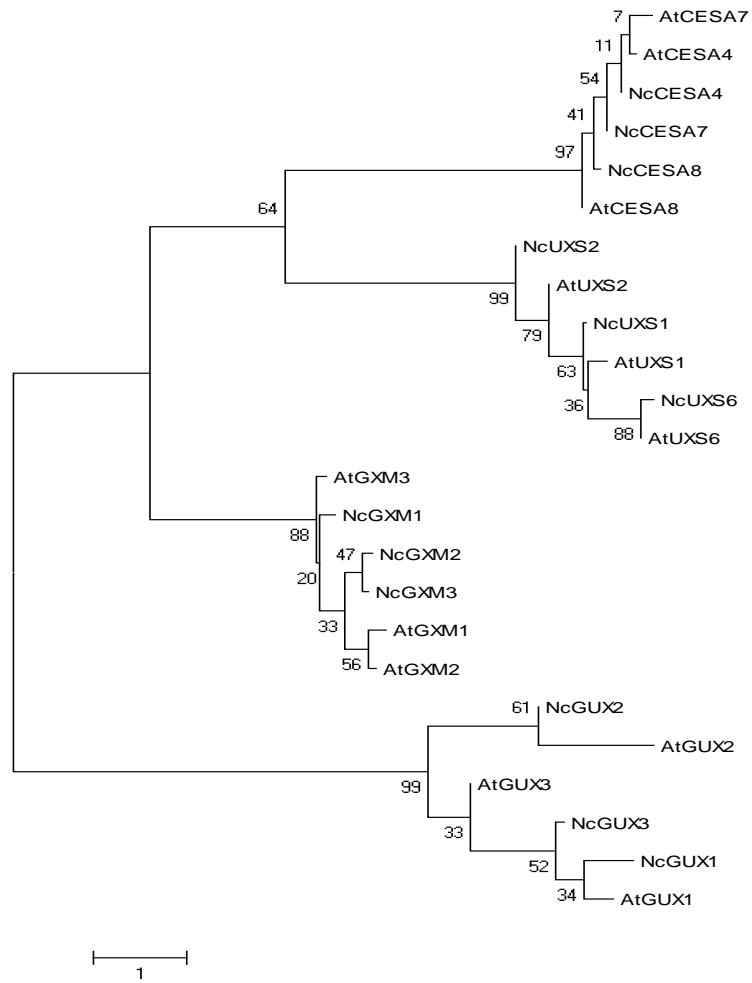

Figure S2 Phylogeny tree of genes profiled.

Supplement: Figure S2 — Phylogeny tree of genes profiled. [file Figure_2.PDF]
